# Central nervous system embryonal tumors with EWSR1-PLAGL1 rearrangements reclassified as INI-1 deficient tumors at relapse

**DOI:** 10.1007/s11060-024-04667-6

**Published:** 2024-04-19

**Authors:** Kevin J. Bielamowicz, Mary Beth Littrell, Gregory W. Albert, Lora S. Parker, Sateesh Jayappa, Kenneth Aldape, Murat Gokden

**Affiliations:** 1 Division of Pediatrics, The University of Arkansas for Medical Sciences (UAMS), 1 Children’s Way Slot 512-10, 72223 Little Rock, AR USA; 2 Section of Pediatric Hematology/Oncology, UAMS, Little Rock, AR USA; 3 Division of Pathology, UAMS, Little Rock, AR USA; 4 Department of Neurosurgery, UAMS, Little Rock, AR USA; 5 Division of Radiology, UAMS, Little Rock, AR USA; 6 Arkansas Children’s Hospital, Little Rock, AR USA; 7 Division of Neurosurgery, ACH, Little Rock, AR USA; 8 Laboratory of Pathology, National Cancer Institute, Bethesda, MD USA

**Keywords:** Brain tumor, Embryonal tumors, EWSR1-PLAGL1 rearrangements, Atypical teratoid rhabdoid tumors, INI-1 deficient tumors

## Abstract

**Purpose:**

Central nervous system (CNS) embryonal tumors are a diverse group of malignant tumors typically affecting pediatric patients that recently have been better defined, and this paper describes evolution of a unique type of embryonal tumor at relapse.

**Methods:**

Two pediatric patients with CNS embryonal tumors with EWSR1-PLAGL1 rearrangements treated at Arkansas Children’s Hospital with histopathologic and molecular data are described.

**Results:**

These two patients at diagnosis were classified as CNS embryonal tumors with EWSR1-PLAGL1 rearrangements based on histologic appearance and molecular data. At relapse both patient’s disease was reclassified as atypical teratoid rhabdoid tumor (ATRT) based on loss of INI-1, presence of SMARCB1 alterations, and methylation profiling results.

**Conclusion:**

CNS embryonal tumors with EWSR1-PLAGL1 rearrangements acquire or include a population of cells with SMARCB1 alterations that are the component that predominate at relapse, suggesting treatment aimed at this disease component at diagnosis should be considered.

## Introduction

Central nervous system embryonal tumors (CNS-ETs) are highly aggressive, poorly differentiated malignancies occurring predominantly in young children but also affecting adolescents and adults. While historically categorized uniformly based on histology which includes small, poorly differentiated or undifferentiated embryonal cells, CNS-ETs have been shown to encompass several distinct entities based on molecular profiling, most recently in the 2021 WHO CNS Classification (CNS5) criteria [10, 15].

Atypical teratoid rhabdoid tumors (ATRT) are a type of CNS-ET that more commonly occurs in infants and carry a poor prognosis [2, 7, 10, 12, 19]. ATRT are molecularly defined by alterations in the SWI/SNF chromatin remodeling complex members SMARCB1 and SMARCA4 [5, 8]. More recently, ATRT have been shown to comprise different genetic subgroups. Three clinicopathologic subtypes of ATRT have been recognized by transcriptome and DNS methylation profiling studies [5, 7].

Historically, CNS-ETs and ATRT have been treated similarly [2–4, 6, 7, 12, 16–19]. Patients at the age where craniospinal radiation is thought to carry reasonable neurocognitive outcomes can be treated with craniospinal radiation followed by maintenance chemotherapy. Those who are younger often receive chemotherapy potentially intensified to high dose chemo with autologous stem cell rescue and focal radiation, intrathecal chemotherapy, or other strategies. With the growing knowledge regarding genetic subtypes, the ability to delineate which genetic subtypes may benefit from particular traditional treatment components and future targeted and novel therapies is essential.

EWSR1 gene rearrangements have been reported in CNS neoplasms [9, 11]; Roosen, Ode, Bunt, & Kool [13],; Sturm et al [15]. Some of these EWSR1 rearrangements are fusions with the PLAGL1 gene, and other PLAGL1 gene rearrangements in CNS neoplasms have also been described [14]. One case of an INI-1 deficient EWSR1-PLAGL1 CNS embryonal tumor has been described, raising the question as to whether this entity overlaps with ATRT [11].

In this study we describe two patients with initial diagnoses of CNS-ETs with EWSR1-PLAGL1 gene fusions whose definition changed at relapse which could affect the way this rare subtype is approached therapeutically in the future.

## Methods

Two pediatric patients with CNS-ETs with EWSR1-PLAGL1 rearrangements treated at Arkansas Children’s Hospital are described. The research project was submitted to and approved by the institutional review board (IRB) of the University of Arkansas for Medical Sciences (UAMS) and was determined to be exempt from family consent and full IRB review per UAMS IRB policy. The patient’s case data was obtained from the electronic medical records at Arkansas Children’s Hospital. The data underlying this article are available in the article and in its online supplementary material. Tumor sequencing was performed by Tempus xT 648 gene targeted sequencing panel, including both somatic and germline sequencing (www.tempus.com). Methylation profiling was performed by the Laboratory of Pathology at the National Cancer Institute/National Institutes of Health, using the Heidelberg version 12.5 classifier (https://www.molecularneuropathology.org/mnp/).

## Patient cases

### Case 1

**Fig. 1:**
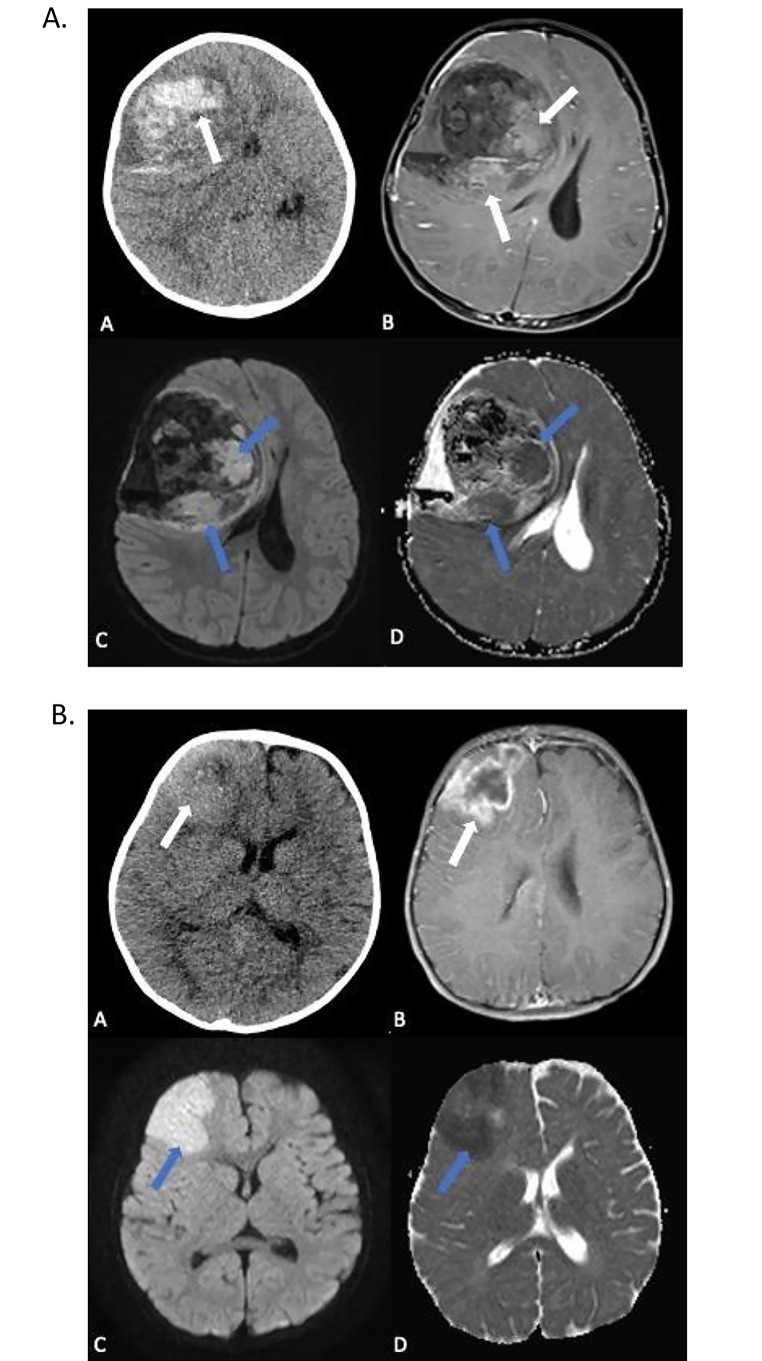
**a**: Initial imaging studies for patient 1. (**A**) Non-contrast axial CT scan of head shows a large hemorrhagic right frontal lobe mass lesion with mass effect and midline shift (white arrow). (**B**) Axial post-contrast T1-weighted MR image demonstrates right frontal heterogenous mass with enhancing soft tissue components (white arrows). (**C**) Diffusion weighted image and (**D**) ADC demonstrate restricted diffusion in the areas of enhancing soft tissue components, appearing bright on diffusion and dark on ADC (blue arrows) **b**. Initial imaging studies for patient 2. (**A**) Axial non-contrast CT scan of the head shows an ill-defined hyperdense mass with punctate calcifications and a small necrotic area (white arrow). (**B**) Postcontrast T1-weighted MR image demonstrates heterogeneously enhancing frontal lobe mass with necrotic area (white arrow). (**C**) DWI and (**D**) ADC images show restricted diffusion indicating highly cellular components (blue arrows)

The patient presented at 14 months of age with vomiting and decreased alertness. Imaging showed a right frontal brain mass (Fig. 1). She had an up front gross total resection (GTR) of the tumor.

**Fig. 2:**
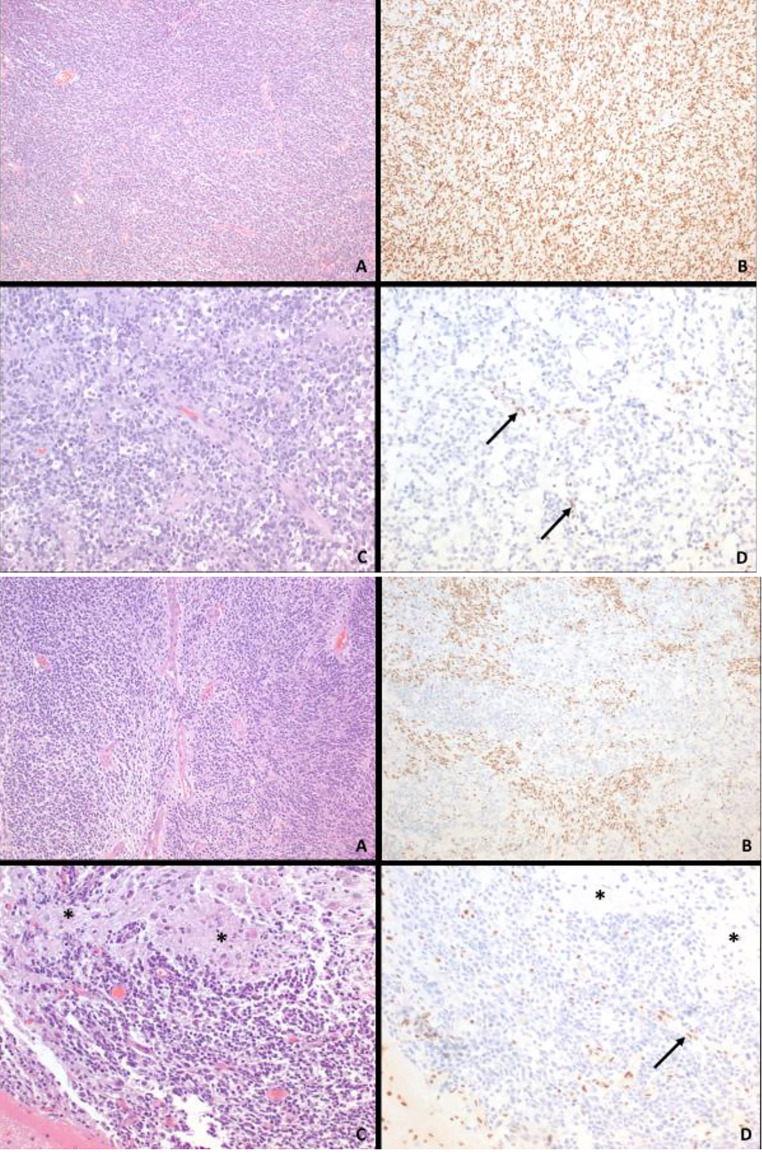
**a**: Patient 1 pathology: A. Representative histology of the embryonal neoplasm and corresponding INI1 immunohistochemistry (IHC). **A**. Initial resection specimen and **B**. Corresponding INI-1 IHC showing retained nuclear expression. **C**. Both recurrent neoplasms had similar features. **D**. Nuclear INI1 expression is entirely lost in the neoplastic cells, with blood vessels (arrows) serving as internal control with retained expression. (Original magnifications: A-D 100x) **b**: Patient 2 pathology: Representative histology of the embryonal neoplasm and corresponding INI1 immunohistochemistry (IHC). **A**. Initial resection specimen and **B**. Corresponding INI-1 IHC showing a mosaic pattern with alternation areas of retained and lost nuclear expression. **C**. Recurrent neoplasm (center, top right) growing on the surface of the brain (bottom left). Focal areas of maturation are also present (*). **D**. Nuclear INI1 expression is entirely lost in the neoplastic cells, including in the areas of maturation (*), with brain tissue (bottom left) and blood vessels (arrow) serving as internal control with retained expression. (Original magnifications: A-D 100x)

The surgical specimen showed a small, blue cell neoplasm with brisk mitotic activity and frequent apoptotic cells. Immunohistochemically, the primary neoplasm was negative for CD99, EMA, and desmin and Neu-N, with rare cells being weakly positive for S-100 protein, neurofilament protein, GFAP and synaptophysin. Olig-2 was positive in many neoplastic nuclei. Ki-67 proliferation index was 25–30%. The tumor was negative for CD99. P53 was overexpressed in a significant subset of nuclei. ATRX staining suggested a wildtype pattern. The tumor showed retained INI-1 (Fig. 2) and BRG-1 expressions. The tumor stained negative for ALK, BCOR, and NUTM1. It was negative for C19MC, BCOR, CIC, and MN1 by FISH. Targeted sequencing showed an EWSR1-PLAGL1 rearrangement as well as overexpression of EZH2, FGFR2, HRAS, and TOP2A (Table 1). No somatic tp53 mutation or alteration was identified. No pathogenic germline alterations were identified. Methylation profiling and the final integrated diagnosis categorized the disease as a Neuroepithelial tumor, PLAGL1 fused (Table 1).

She completed chemotherapy treating according to ACNS0334 with methotrexate. End of therapy scans showed no evidence of residual disease. Imaging 3 months after the completion of therapy showed two nodules at the primary tumor site. Staged resection of these nodules were confirmed to be a recurrent embryonal tumor and a second GTR was achieved. She then received focal proton therapy. MRI 7 months post radiation showed local recurrence and she had resection of the recurrent tumor. She started adjuvant irinotecan/temozolomide/Avastin and completed 7 weeks of therapy before having further symptomatic recurrence. She then received high dose craniospinal radiation with a boost to the recurrent site. Post CSI scans showed no concern for distant metastasis but possible progression at the primary site.

**Table 1:** Highlights of both patient cases are presented with respect to histopathologic findings, targeted sequencing results, methylation profiling classification, and the integrated diagnosis. VAF - variant allele frequency

	Tumor Sample	Histopathology	Targeted Sequencing	Methylation Profiling Classification	Integrated Diagnosis
Case 1	Diagnostic	-Synaptophysin positive -Rhabdoid cells negative -INI1 retained -Brg-1 retained -C19MC region chromosome 19 not amplified	-EWSR1-PLAGL1 rearrangement -Overexpression of EZH2, FGFR2, HRAS, and TOP2A	Neuroepithelial tumor, PLAGL1 fused	Neuroepithelial tumor, PLAGL1 fused
	Relapse	Similar to Diagnostic specimen, with INI1 loss	-SMARCB1 copy number loss -EWSR1-PLAGL1 rearrangement -Overexpression of EZH2, FGFR2, HRAS, TOP2A	Atypical Teratoid/Rhabdoid Tumor, subclass SHH	Atypical Teratoid/Rhabdoid Tumor, SHH subgroup, WHO grade 4
Case 2	Diagnostic	-Synaptophysin positive -Rhabdoid cells negative -INI1 retained -Brg-1 retained -C19MC region chromosome 19 not amplified	-EWSR1-PLAGL1 rearrangement -Overexpression of CCND1, TOP2A, and HRAS	No match	CNS Embryonal Tumor with EWSR1-PLAGL1 rearrangement
	Relapse	Similar to diagnostic sample, with INI1 loss	-EWSR1-PLAGL1 rearrangement -SMARCB1 alteration p.r53 stop gain LOF (14.5% VAF); BTG1 copy number loss -Overexpression of CCNE1, KRAS, HRAS	Atypical Teratoid/Rhabdoid Tumor, SHH Subtype	Neuroepithelial tumor, PLAGL1 fusion-positive, with acquired SMARCB1 mutation.

Recurrent tumors showed a pathogenic SMARCB1 copy number loss on repeat sequencing more consistent with ATRT (not present on the initial diagnostic tumor specimen) while retaining the molecular features of the original tumor (Table 1). Immunohistochemical stain performed for INI-1 on recurrent tumor specimen showed loss of nuclear expression (Fig. 2). Repeat methylation profiling classified the tumor as ATRT, SHH subtype. She then began treatment with single agent tazemetostat. Scans showed further progression of disease 2 months after beginning this therapy. Family elected against any further disease directed treatment and the patient died of disease 33 months after diagnosis.

### Case 2

Patient 2 was a male who presented at the age of 21 months with new onset seizures which included abnormal hand movements, altered consciousness, and eventually generalized tonic-clonic movements. Imaging showed a localized right frontal tumor (Fig. 1). He had an upfront gross total resection of the tumor.

Histologically the tumor showed a small blue cell morphology. The neoplastic cells had round to spindled nuclei with coarse chromatin pattern. Nucleoli and cytoplasms are inconspicuous, but there were areas where the cellularity was loose in a patchy neuropil-like background. Frequent mitotic figures, apoptotic debris, areas of necrosis and calcification were also seen. There was infiltration of the leptomeninges, in one area, filling up a sulcus. Several foci showed prominent vascular proliferation. A few foci showed cells with prominent, eosinophilic cytoplasm and an open chromatin, i.e., somewhat rhabdoid appearance. Immunohistochemically, there was variable and patchy positivity for glial markers (GFAP, olig-2, S-100 protein) and neuronal markers (synaptophysin, neurofilament protein). Vimentin was diffusely and strongly positive. P53 was strongly overexpressed in a large subset of cells. Ki-67 proliferation index was 40–50% in many active areas. INI-1 expression was largely retained (including in the small groups of rhabdoid-appearing cells) but showed a mosaic pattern of loss in several foci. BRG-1 and ATRX each showed retained nuclear expression (Fig. 2). EMA weakly stained occasional cells. Pancytokeratin was negative.

Targeted sequencing showed a EWSR1-PLAGL1 rearrangement as well as overexpression of CCND1, TOP2A, and HRAS (Table 1). No somatic tp53 gene mutation or alteration was identified. No somatic germline alterations were identified. It was commented that the significance of this rearrangement, as well as that of the focal loss of INI-1 nuclear expression, was not clear, and that the findings are not diagnostic of ATRT or Ewing Sarcoma, or other defined neoplasms characterized by EWSR1 rearrangements. Methylation profiling did not identify a class match. The integrated diagnosis was signed out as CNS Embryonal Tumor with EWSR1-PLAGL1 rearrangement.

He completed chemotherapy treating according to ACNS0334 without methotrexate. End of therapy scans showed areas of concern at the previous surgical resection site concerning for recurrence. He had a GTR of the new tumor and pathology was consistent with malignant recurrence with similar histologic findings, but now with INI-1 loss (Fig. 2). Targeted sequencing of the recurrent specimen showed the same EWSR1-PLAGL1 rearrangement, but also with a SMARCB1 mutation (p.r53 stop gain LOF, 14.5% variant allele frequency) and BTG1 copy number loss. There was also overexpression of CCNE1, KRAS, and HRAS genes. Methylation profiling was suggestive of ATRT and this was the integrated diagnosis of the recurrent specimen (Table 1).

He was referred for focal radiation therapy after his second GTR but on pre-radiation planning imaging prior to starting radiation he had rapid metastatic recurrence. He began a modified MEMMAT protocol but had symptomatic and radiographic progression after 8 weeks of treatment. He then began treatment with single agent tazometostat but again had progression of disease after 1 month of treatment. After deciding against any further disease directed treatment he died of disease progression 11 months after diagnosis.

## Discussion

This case series presents the clinical, pathologic, and molecular data for two young patients who at presentation were diagnosed with CNS embryonal tumors with EWSR1-PLAGL1 rearrangement. Upon recurrence specimen analyzed for both patients had SMARCB1 alterations not identified at diagnosis while still retaining the EWSR1-PLAGL1 rearrangement. On immunohistochemistry both patients had INI-1 loss on recurrence which was not present at diagnosis. While not formally recognized by the WHO CNS5 criteria, patients with CNS embryonal tumors and PLAGL1 rearrangements have been described.

These patients are typically young pediatric patients with a median age of 6 years with tumors in the supratentorial region [14]. One report also identified a case of an INI-1 deficient tumor with and EWSR1-PLAGL1 rearrangement [11]. The SMARCB1 rearrangement could be acquired during treatment, but since the second patient had patchy INI-1 loss on IHC it could also be that these tumors are more susceptible to developing SMARCB1 alterations and a subset of cells in these tumors have this alteration at diagnosis and are the component most resistant to treatment. The fact that the disease for these two patients were assigned a different methylation class upon development of the SMARCB1 alterations at recurrence is notable. Change of DNA methylation class between primary and recurrent tumor is very unusual for brain tumors and this may represent one of the few exceptions and needs to be verified in larger cohort of patients. Our patients progressed rapidly once the tumor primarily had INI-1 deficiency, and the second patient which showed some INI-1 loss at diagnosis by IHC had a more rapid clinical progression, and this focal losses of nuclear INI-1 expression in an ET may be a clue to the diagnosis and nature of these neoplasms. The progression was primary early and local at the presenting primary tumor site at first relapse, so early focal radiation may be beneficial.

While the findings of development and/or progression of INI-1 deficient malignancy in these patients needs to be validated in a larger cohort, this may be difficult in a very rare disease. These patients could potentially benefit from strategies that have resulted in improvement in outcomes in ATRT including high dose chemotherapy with autologous stem cell rescue and early focal radiation [2, 4, 12, 19], incorporation of intrathecal chemotherapy at diagnosis [2, 4], high dose early craniospinal radiation in older patients [16], and newer therapies that have shown promise for INI-1 deficient tumors [1]. Consideration should be given to including these patients in clinical trials for patients with ATRT at diagnosis.

## Data Availability

No datasets were generated or analysed during the current study.
